# Relational continuity in nurse–patient contact on hospital wards: An explanatory sequential mixed-methods study

**DOI:** 10.1016/j.ijnsa.2026.100560

**Published:** 2026-05-25

**Authors:** Heleen van Erp, Jet Bussemaker, Meralda Slager, Janneke de Man-van Ginkel

**Affiliations:** a Leiden University Medical Center, Nursing Science, Albinusdreef 2, 2333 ZA Leiden, the Netherlands; b Haga Teaching Hospital, Els Borst-Eilersplein 275, 2545 AA The Hague, the Netherlands; c Leiden University, Institute of Public Administration, Faculty Governance and Global Affairs, Turfmarkt 99, 2511 DP The Hague, the Netherlands; d Leiden University Medical Center, Public Health and Primary Care, Hippocratespad 21, 2333 ZD Leiden, the Netherlands; e Avans University of Applied Sciences, Hogeschoollaan 1, 4818 CR Breda, the Netherlands

**Keywords:** Emotional demands, Mixed methods research, Moral distress, Nurse–patient relations, Nursing staff, hospital, Relational continuity, Relational regulation

## Abstract

**Background:**

Rising care complexity, shorter hospital stays, and nursing shortages limit opportunities for recurrent nurse–patient contact, the structural foundation of relational continuity. While this contact is essential for relationship formation, its frequency in hospital wards remains persistently low. Understanding the mechanisms and contextual factors that shape contact patterns is therefore crucial to support relational continuity in nursing practice.

**Objective:**

To explore relational continuity in nurse–patient contact on general wards and to examine nurses’ perspectives on the mechanisms and contextual factors underlying current practices.

**Design:**

Mixed-methods explanatory sequential design.

**Setting:**

General nursing wards in a teaching hospital in the Netherlands.

**Participants:**

A total of 126 patient records were included in the quantitative component; three focus groups involving 11 nurses were conducted in the qualitative component.

**Methods:**

In the quantitative phase, authorship of reports in patient records was used as an indicator of nurse–patient contact. Descriptive statistics were then applied to assess the frequency of recurrent contact as a proxy for relational continuity across seven wards. Subsequently, these observed contact patterns were discussed in focus groups with nurses. Qualitative data were analyzed using thematic analysis to identify contributing mechanisms and contextual factors. Integration of both strands occurred through an integrated interpretation, linking observed contact patterns to nurses’ perspectives.

**Results:**

Recurrent nurse–patient contact was limited. During an average hospital stay of 6.5 days, authored reports were written by a mean of 11.4 different nurses, with 82.7% representing only a first or second report by the same nurse. While nurses typically maintained contact for two to three shifts, they often withdrew thereafter. This observed contact pattern was shaped by an interplay between organizational constraints and mechanisms of relational regulation, whereby pragmatic rationalizations enabled nurses to mitigate moral distress when professional values regarding relational continuity were compromised.

**Discussion:**

These findings indicate that relational continuity is not merely an organizational challenge but is also shaped by how nurses navigate their contact with patients. Workforce policies targeting organizational factors in isolation are likely to fall short if they overlook the relational regulation that leads nurses to withdraw when contact becomes emotionally demanding. Effective strategies should therefore integrate support for these emotional demands with organizational adjustments, thereby enabling nurses to better realize professional values in daily practice.

▓

**What is already known**

• Relational continuity is a fundamental nursing component, underpinning informational and management continuity in hospital care.
• Recurrent nurse–patient contact is a structural precondition for developing nurse–patient relationships.
• Achieving relational continuity remains a persistent challenge, often attributed to organizational constraints.

**What this paper adds**

• Recurrent nurse–patient contact typically declines after two to three shifts.
• Nurses’ withdrawal is shaped by the interplay between organizational constraints and the emotional demands of sustained contact.
• Pragmatic rationalizations of contact disruptions help mitigate moral distress.

Alt-text: Unlabelled box dummy alt text

## 1 Background

The global rise in chronic illness and population aging is increasing the demand for inpatient care (Organisation for Economic Co-operation and Development, 2024; World Health Organization, 2020). Simultaneously, hospital stays are becoming shorter due to financial pressures and medical-technological advances (Golinelli et al., 2024). These trends place additional strain on hospital nursing, which already faces a projected global shortage of 5.7 million nurses by 2030 (OECD, 2024). As patients require more complex care in less time and with fewer staff, ensuring continuity of nursing care is critical for delivering safe, efficient, and person-centered care.

Continuity of care is commonly conceptualized as comprising three interrelated types: relational, informational, and management continuity (Haggerty et al., 2003). Among these, relational continuity, described as an ongoing therapeutic relationship between patient and one or more providers (Haggerty et al., 2003), is considered foundational, as it supports both communication (informational continuity) and care coordination (management continuity) (Bahr and Weiss, 2019; Waibel et al., 2011). In nursing, this relational dimension is recognized as the core element of the 'Fundamentals of Care' framework, representing the professional relationship that enables the integration of physical, psychosocial, and relational needs, and serves as the basis for quality nursing care (Feo et al., 2017). These relationships involve a process of "getting to know" the patient, which builds through recurrent contact over time (Allande-Cussó et al., 2022; Zolnierek, 2014). As relationships develop, interpersonal affect and shared feelings emerge (Allande-Cussó et al., 2022; Hobbs, 2009). These shared feelings are an important source of nursing knowledge, referred to as aesthetic knowledge, which concerns “knowing how to” be with a patient. This process humanizes care and positively impacts the nurse–patient relationship (Allande-Cussó et al., 2022; Carper, 1978).

Relational continuity is structurally underpinned by recurrent contact with the same professional, providing patients with predictability and coherence (Bahr and Weiss, 2019; Reid et al., 2002). This concept has a strong tradition in medical research, particularly regarding longitudinal care. In this domain, the benefits of continued contact, often referred to as consistency of personnel, are well-documented, specifically regarding patient satisfaction, trust, and relationship development (Dyer et al., 2022; Pereira Gray et al., 2018; Van Walraven et al., 2010). In hospital nursing, relational continuity takes on a distinct character defined by intensity and 24-hour presence rather than years of contact. Although a hospital stay is relatively brief and episodic, the constant proximity of nursing staff makes relational continuity an important component of the care process. Since recurrent contact is considered a prerequisite for building a nurse–patient relationship (Allande-Cussó et al., 2022; Zolnierek, 2014), it can be viewed as the structural foundation of relational continuity in acute care settings.

Despite its potential value, achieving relational continuity in modern hospital nursing is increasingly difficult. Nurses rarely work five or more consecutive shifts (Martins et al., 2025), and factors such as specialization, part-time employment, reliance on temporary staff, high administrative burdens, and shorter lengths of stay contribute to the fragmentation of inpatient care (Organisation for Economic Co-operation and Development, 2022; Yakusheva et al., 2017; Zolnierek, 2014). Together, these constraints reduce opportunities for recurrent nurse–patient contact, undermining the foundation for relationship building.

Nursing research on the clinical outcomes of relational continuity, such as pressure ulcers, functional decline, or readmissions, has yielded mixed and often inconclusive results (Bahr et al., 2020; Ji et al., 2023; Stifter et al., 2015; Tonkikh et al., 2020; Yakusheva et al., 2017). However, the validity of these clinical outcomes is difficult to interpret as long as the actual patterns of recurrent contact, the building blocks of relational continuity, remain unexamined in daily nursing practice. Without first understanding these contact patterns and the drivers that shape them, researchers cannot properly interpret how relational continuity impacts clinical outcomes. Therefore, gaining insight into how these patterns emerge and why they persist is a necessary prerequisite. Most notably, there is a dearth of research that integrates objective data on contact frequency with the professional perspectives of nurses who must navigate the fragmented environment of modern hospital care.

**Aim** The aim of this study was to explore relational continuity in nurse–patient contact on general wards and to examine nurses’ perspectives on the mechanisms and contextual factors underlying current practices.

To achieve this aim, the study addressed three interconnected research questions (RQ):

• RQ1: What is the frequency of recurrent contact, using authored reports as an indicator for nurse–patient contact, to indicate the extent of relational continuity on general wards?
• RQ2: How do nurses perceive the extent of relational continuity as reflected in the observed contact patterns, and what mechanisms and contextual factors do they identify as contributing to these patterns?
• RQ3: How do nurses’ perspectives and the identified mechanisms and contextual factors explain the observed patterns of recurrent nurse–patient contact?

## 2 Methods

### 2.1 Design and setting

This study used an explanatory sequential mixed-methods design, grounded in pragmatism, with the qualitative strand adopting an interpretivist stance (Creswell and Plano Clark, 2018).

First, we mapped patterns of recurrent nurse–patient contact to assess relational continuity (RQ1).

Subsequently, these results were presented via a visual summary to nurses in focus groups to explore the underlying reasons for these patterns (RQ2). Data integration occurred at the methods level by using quantitative results to inform qualitative data collection, and at the interpretation level by merging both data sources to explain how nurses' perspectives, underlying mechanisms, and contextual factors shape observed contact patterns (RQ3).

The study was conducted on all seven general wards of a large Dutch teaching hospital (21–42 beds per ward). Nurses work 8-hour shifts; nurse-to-patient ratios are 1:4–5 during day shifts, 1:8 in the evening, and 1:11 at night. Most nurses work between 24 and 36 h per week. Under the hospital’s individual patient assignment model, nurses are responsible for both direct patient care and clinical reporting for an allocated group of patients per shift. The electronic health record (EHR) system automatically links each authored report to the nurse’s unique login credentials.

### 2.2 Data collection

***The first, quantitative, phase*** involved a retrospective record review (n = 126). Relational continuity was operationalized as recurrent contact, calculated by tracking the frequency with which the patient's reports were authored by the same nurse. To quantify the patient’s actual exposure to different nurses during their stay, authored reports served as an indicator of nurse–patient contact, while the frequency of contact functioned as a proxy for relational continuity. This phase provided a mapping of contact patterns to inform the focus groups, rather than aiming for statistical generalizability.

Per-shift authorship data were extracted from the electronic records of discharged patients by three trained researchers using a standardized protocol and entered into a pilot-tested database in Castor EDC. Extracted variables included patient demographics (age, gender, and partial postcode), admission details (dates, ward, and room number), and authorship for every morning, evening, and night shift. If multiple nurses authored a report in a single shift, all were recorded. Nurse identities were pseudonymized using unique nurse study numbers. To ensure traceability to the original records, a separate key list linked the unique Castor study codes to patient identifiers (names and patient identification numbers). Both the nurse and patient key lists were password-protected and stored on a secured institutional drive, separate from the research database.

Records were screened chronologically based on the electronic discharge lists of each participating ward, using a consecutive sampling strategy. Records were eligible if patients were admitted to the same ward for 3–14 days (9–42 shifts); records of deceased patients or those transferred from other wards were excluded. This range reflects standard clinical stays (hospital average: 5.2 days), providing a sufficient window to identify contact patterns while excluding outliers. Missing reports were coded as “999” and excluded from the authorship frequency analyses.

Data were extracted on 21 separate occasions, scheduled across different days of the week and randomly distributed throughout the 2020–2022 period, with a temporary pause during the COVID-19 pandemic due to altered care models.

Sourcing records from seven wards over two years helped mitigate sampling bias, ensuring contact patterns reflected institutional routines rather than department-specific staffing fluctuations or surges in patient acuity. To ensure a consistent process across all wards and time periods, data extraction followed a standardized protocol that was designed by HvE and checked for face validity by the data collectors. A random spot-check of five records showed no discrepancies between the data collectors, confirming inter-rater reliability.

***In the second, qualitative phase***, staff nurses and senior nurses from the wards involved in the record review were invited to participate in focus group discussions via their supervisors. Each focus group was composed of participants from different wards, ensuring only one nurse per ward per session, to promote an open dialogue where reflections on similarities and differences in practices could be exchanged. Senior nurses were specifically included for their dual role (combining clinical shifts with quality and policy responsibilities) to ensure the inclusion of both direct clinical insights and broader organizational perspectives; their role is non-hierarchical.

Based on the relatively homogeneous nature of the sample, three 90-minute focus groups were initially planned (Guest et al., 2017) and conducted between May and June 2023. While the study aimed for a target sample of 21 participants (one nurse per ward for each focus group), turnout was affected by acute clinical workload and last-minute withdrawals. Despite the smaller group sizes, data collection was concluded after the third session, as preliminary analysis confirmed that the data provided sufficient depth and the third focus group yielding no significant new insights.

The focus groups were moderated by the PI (HvE), whose background as a nurse researcher facilitated rapport, while her independent position (not employed at the wards) encouraged an open environment. Each session was supported by a note-taking observer; these were nurse researchers from the same hospital, either internal or external to the participating wards. The combination of the PI’s independent position and the observers’ varying degrees of familiarity with the wards enhanced the contextual depth of the immediate post-session debriefings.

Data collection followed a semi-structured topic guide developed from the study objectives and Phase 1 results (see Appendix A for the full topic guide). The guide was structured around three key domains: (1) structural determinants, such as patient assignment and shift patterns; (2) the evolution of nurse–patient contact across shifts and the 'turning point'; and (3) the influence of team dynamics and ward culture on continuity. These domains were defined to elicit the mechanisms and contextual factors underlying recurrent contact and relational continuity, in relation to RQ2 and RQ3.

Each session began with visual elicitation: a summary of the observed contact patterns was presented to ensure a shared understanding. The PI then prompted the discussion with: “Hearing this, what do you think?” Following initial reactions, targeted probing questions were employed to further explore specific situations, individual experiences, and nurses' perceptions. This approach served a dual purpose. First, it acted as a member check to validate the authorship-based proxy by assessing whether the observed patterns were recognizable to the participants. Second, it triggered discussion on underlying causes and the perceived impact on relational continuity. Sessions were audio-recorded, transcribed verbatim, and pseudonymized.

### 2.3 Data analysis

***Quantitative data*** were analyzed using descriptive statistics in R. For each patient record, the number of unique authors was calculated. These totals were summarized as means and standard deviations (SD) for the total sample and per admission category (short, medium, and long stay).

To quantify contact frequency, we determined for each authorship whether it represented the nurse's first, second, third, or subsequent authorship for that specific patient; these frequencies were expressed as percentages of the total number of authorships.

***Qualitative data***, comprising focus group transcripts and observational notes, were analyzed inductively using thematic analysis (Braun and Clarke, 2006) with ATLAS.ti 24. First, three hospital-affiliated researchers independently read the focus group transcripts and made reflective notes to familiarize themselves with the data. These notes were then discussed within the team to establish a shared initial understanding. Next, each transcript was subjected to independent open coding by two researchers: the first author (PI) coded all three transcripts, while a second researcher coded transcripts 1 and 3, and a third researcher coded transcript 2. The researchers subsequently compared their open codes in joint sessions to develop a shared codebook. Based on this codebook, the PI identified potential themes and developed a thematic map. These were first discussed with the co-researchers to ensure the themes aligned with their findings, and subsequently refined in iterative sessions with the supervision team to provide an 'outsider' perspective and ensure analytical rigor.

**Fig. 1:**
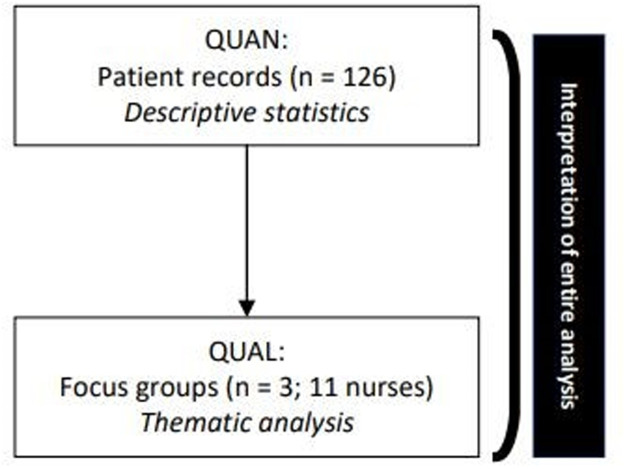
Flowchart of the explanatory sequential research design. This flowchart (adapted from Creswell and Plano Clark, 2018) illustrates the sequential transition from the quantitative (QUAN) phase to the qualitative (QUAL) phase. The vertical arrow signifies the use of quantitative results as direct input for the qualitative strand. The bracket on the right indicates the integration and final interpretation of the entire analysis.

***Data were integrated*** through a narrative approach by weaving the findings together during the interpretation phase (Fetters et al., 2013). This integration was achieved by triangulating the observed contact patterns with the qualitative themes to explore how the quantitative data aligned with the participants' clinical experiences (Fig. 1).

## 3 Results

### 3.1 Phase 1: quantitative contact patterns

A total of 126 patient records from seven general nursing wards were analyzed. Each ward contributed 12–25 records, except for one with a smaller bed capacity, which contributed 8 records. Length of stay averaged 19.1 shifts (approximately 6.5 days), which was slightly above the hospital’s overall mean because admissions shorter than nine shifts were excluded. The sex distribution was evenly balanced (Table 1).

**Table 1:** Characteristics of included records (N = 126).

Ward	n	%
Internal medicine/ geriatrics	24	19.0%
Cardiology/ cardiothoracic surgery	17	13.5%
Pulmonary medicine	12	9.5%
Neurology/ neurosurgery	25	19.8%
Surgery/ traumatology	22	17.5%
Surgery/ gastroenterology	8	6.3%
Oncology/ hematology	18	14.3%
Length of stay		
Short (9–14 shifts)	49	38.8%
Medium (15–23 shifts)	44	34.9%
Long (24–42 shifts)	33	26.2%
Patient’s sex		
Male	63	50%
Female	63	50%

Across 2410 shifts, the individual assignment model was largely followed, with most reports authored by a single nurse (89.9%). However, collaborative documentation was observed in 9.7% of shifts, where two or more nurses authored reports for the same patient. Only a negligible fraction of shifts lacked reports. This resulted in a total of 2643 authorships, serving as the proxy for nurse–patient contact (Table 2).

**Table 2:** Distribution of nursing report authorship per shift (N = 2410 shifts).

Authorship per shift	n	% of reports
Reports authored by 1 nurse	2166	89.9%
Reports authored by multiple nurses (≥2)	233	9.7%
No report documented	11	0.5%
Total	2410 shifts	
	2643 authorships	

**Fig. 2:**
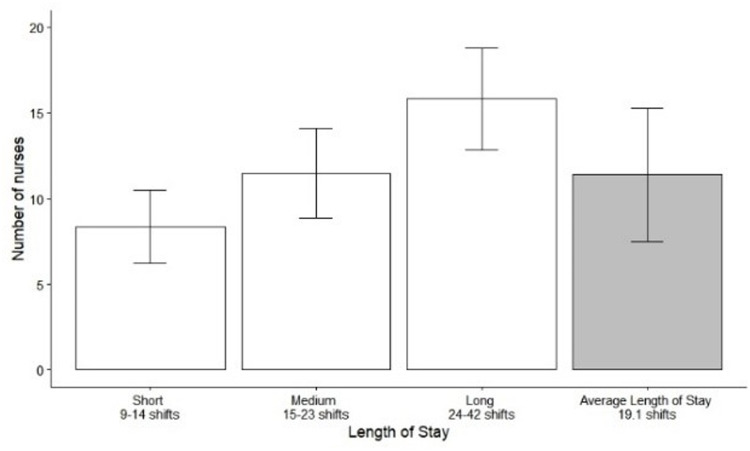
Average number of unique nurses authoring reports per patient admission. Data are categorized by length of stay (LOS): short, medium, and long stays, compared to the overall mean (average). The vertical bars represent the mean number of unique authors, with error bars indicating the standard deviation (SD).

Based on this proxy, an average of 11.4 different nurses authored reports per patient record (SD 3.9) across the mean admission duration of 19.1 shifts. The number of unique authors for patients with short stays averaged 8.3 (SD 2.1), compared with 11.5 (SD 2.6) for medium stays and 15.8 (SD 3.0) for long stays (Fig. 2).

Analysis of the 2643 authorships mapped patterns of recurrent contact. More than half (54.3%) represented a nurse’s first authorship for a specific patient, while 28.3% were a second authorship by the same nurse. Only 17.3% reflected a third to fifth authorship, and sixth or subsequent authorships were rare. Overall, 82.7% of all authorships were accounted for by nurses who authored reports for a specific patient only once or twice during the entire admission (Table 3).

**Table 3:** Frequency of repeated authorship by unique nurses per patient admission (N = 2643 authorships).

Frequency of authorship	n	%	
First report by unique nurse	1436	54.3	82.7%
Second report by same nurse	749	28.3	
Third report by same nurse	314	11.9	17.3%
Fourth report by same nurse	107	4.0	
Fifth report by same nurse	34	1.3	
Sixth report by same nurse	3	0.1	

### 3.2 Phase 2: qualitative perspectives on relational continuity

Eleven nurses, representing six of the seven wards included in the record study, participated in three focus group sessions (group sizes: 4, 3, and 4). Despite the small groups, these sessions yielded rich data. The diversity of formal roles (six senior nurses and five staff nurses) and clinical experience (ranging from 1.5 to 24 years) fostered vivid interaction. This allowed for an exchange where, for instance, the organizational insights of a senior nurse complemented the frontline perspective of a staff nurse. Discussions proceeded in a constructive atmosphere, with participants reporting a positive experience and interest in hearing their colleagues’ perspectives. Notably, although the initial questions centered on the quantitative results, all groups spontaneously expanded the discussion to relationship-building. Participant characteristics are summarized in Table 4.

**Table 4:** Characteristics participants focus groups.

Focus group	Nurses’code	Ward	Years experience	Gender	Function
1	01	Cardiology/ cardiothoracic surgery	17	F	Senior nurse*
	02	Pulmonary medicine	8	M	Palliative staff nurse
	03	Neurology/ neurosurgery	5	F	Staff nurse
	04	Oncology/ hematology	4	F	Senior* oncology nurse
2	05	Surgery/ gastroenterology	9	F	Senior nurse*
	06	Cardiology/ cardiothoracic surgery	3	F	Senior nurse*
	07	Pulmonary medicine	17	F	Palliative staff nurse
3	08	Neurology/ neurosurgery	1,5	F	Staff nurse
	09	Surgery/ traumatology	15	F	Staff nurse
	10	Oncology/ hematology	20	F	Senior nurse*
	11	Pulmonary medicine	24	M	Senior nurse*
		* Combine clinical shifts with quality and policy responsibilities			

The thematic analysis identified five key themes that provide insight into the contact patterns observed in Phase 1. These themes and their illustrative quotes are summarized in Table 5. In the following sections, each theme is explored in detail and integrated with the quantitative results to provide a comprehensive understanding of relational continuity in the participating wards.

**Table 5:** Themes and illustrative quotes.

	Theme	Illustrative quote *(nurses’ code, focus group number)*
1	Recognition of the quantitative patterns	“That didn't really surprise me, actually. There's so much turnover, and in terms of personnel, too*” (Nurse 09, FG 3)*
2	Beliefs regarding recurrent contact	“Well, in itself, …, the number one priority [in patient assignment] is definitely that we want to maintain that continuity to some extent. And the people who were there yesterday, we will leave them with the same patients. But then the question arises: who is authorized [to deliver all necessary care to this patient] and who is not? Does anyone need to do something for their training? Is there anything that is too complex for the nurse who was there today?” *(Nurse 01, FG 1)*
3	Perceived consequences of continuity	“I think that, I think that it's also nice for us, because you know the patient, you know what a patient has, you know how they think, how, what they want. That's nice, and it's also nice for patients…” *(Nurse 11, FG 3)*
4	Navigating the emotional demands of continuity	“Well, yes, it varies from patient to patient, of course, but with some patients you joke around, you laugh, and I'm much more myself, as I might be with my own family, …, but in any case, I'm more open and not just focused on the clinical picture, but also, um, trying to put someone at ease. And if I notice that someone is very closed off, then I only do what is necessary, so to speak. So then - then I think: okay, if there's no response, then - I'm not really concerned with, um [thinks], yes - putting someone at ease outside of the illness and the hospital stay. Because that just becomes very difficult, I think. Yes.” *(Nurse 05, FG 2)*
5	Disruptions in continuity	“But we do have the agreement that if someone is assigned there—as in your team as well—they will be there again the next day, unless they feel they cannot cope. [Yes, that’s right *(Nurse 07, FG 2)*]. In that case, they are assigned somewhere else.” *(Nurse 06, FG 2)*

Note: All quotes were originally spoken in Dutch and have been translated into English by the research team, preserving the original meaning as closely as possible.

#### 3.2.1 Theme 1: recognition of the quantitative patterns

Nurses participating in the focus groups validated that the nurse who authors the report is almost always the one who provided the care. This confirms that authorship patterns provide a recognizable mapping of actual bedside encounters. In line with this, nurses recognized the limited frequency of repeated authorship (Table 3) as an accurate reflection of their clinical reality. They further pointed out that report authorship is influenced by patient assignment systems. In wards where, for instance, two nurses are assigned to eight patients, there is a higher probability that multiple nurses will contribute to the documentation than in systems where one nurse is assigned to four patients, the latter often leading to a less fragmented pattern. Furthermore, participants noted that assignment routines ranged from prospective planning for an entire week to shorter-term scheduling for the subsequent shift. The responsibility for these assignments also varied, being handled by nurse managers, shift leaders, or through mutual decision-making within the team. Regardless of the system used or the actors involved, frequent changes in assignments occurred across all wards, as further elaborated in Theme 5.

#### 3.2.2 Theme 2: beliefs regarding recurrent contact

This recognition of limited recurrent contact in their daily work led to discussions about the underlying beliefs regarding what continuity should ideally look like. Across all focus groups, nurses expressed that the same nurse should continue to care for the same patient as an important principle in patient assignment decisions. Given this stated professional value, one might expect higher levels of continuity; however, this ideal contrasts with the actual frequency of recurrent contacts (Table 3). The participants themselves reflected this discrepancy through the use of cautious qualifiers such as “in principle,” “we try to,” or “if it works out.” This suggests that while continuity is held as a professional value, it is not consistently achieved in daily practice.

#### 3.2.3 Theme 3: perceived consequences of continuity

These beliefs were closely linked to participants’ perceptions of how continuity affects patients and nurses, highlighting its benefits not only for patients and families but also for themselves and the nursing process. They described how recurrent contact strengthens the nurse–patient relationship, increases patient comfort, reassures relatives, and facilitates more efficient care processes. Familiarity with the patient's situation allowed for more coherent care and improved collaboration with other disciplines. Nurses noted that during a second or third shift, interactions were often smoother and a deeper connection developed. However, the quantitative results (Table 3) show that such frequency is rare, with nurses seldom authoring reports for the same patient more than twice.

Participants also noted that patients appreciated seeing the same nurse again, frequently asking if they would return the next day or inquiring about nurses from previous shifts. They shared that patients expressed pleasure at seeing a familiar face and disappointment when continuity was disrupted, noting that these connections made the stay more pleasant and allowed patients to feel more in control. Nurses believed that patients experienced greater confidence and security, felt more understood, and expressed gratitude and appreciation more often. They noticed that some patients remembered individual nurses long after discharge. In contrast, they believed that lack of continuity leads to a sense of uncertainty among patients, that they struggle with having to rebuild a connection over and over again, and that patients wonder if what is set in motion will continue the next day. Nurses think that causes the patient additional stress. Interestingly, participants also highlighted certain advantages of discontinuity, such as the introduction of a fresh perspective or the opportunity for patients to connect with another nurse if rapport was lacking.

Despite acknowledging these impacts, nurses often downplayed the professional nature of their relational work, describing social interactions as “just a quick chat” or “something you just do,” rather than as intentional professional acts. This points to a paradox: although nurses attribute clear clinical and social benefits to recurrent contact, these values are not reflected in a higher actual frequency of recurrent contacts (Table 3).

#### 3.2.4 Theme 4: navigating the emotional demands of continuity

Although participants valued the benefits of recurrent contact, they also reported that they regularly requested changes in patient assignments. Motivation to maintain contact in subsequent shifts was reinforced by positive experiences when a “click” developed. Conversely, a tendency to withdraw, especially among less experienced nurses, arose when contacts were either perceived as “too close” or, by contrast, when rapport was lacking. These experiences revealed a delicate balance: while recurrent contacts were appreciated for creating human bonds, they could also rapidly become emotionally taxing. When the emotional energy required to sustain contact became too demanding, the need for withdrawal outweighed the perceived benefits of continuity. Consequently, the low frequency of recurrent contacts identified in Phase 1 (Table 3) may reflect a protective mechanism for nurses navigating these emotionally taxing relationships.

#### 3.2.5 Theme 5: disruptions in continuity

The value nurses place on relational continuity contrasts with a routine tolerance for frequent disruptions in practice, which rarely triggered team discussion or apparent internal conflict. Reasons for the disruptions fell into two main categories: organizational factors and personal considerations.

Nurses perceived unit-level organizational reasons as unavoidable and even logical. These were mainly linked to nursing processes or associated with advantages for staff. Examples included scheduling constraints (e.g., rotating shifts, staff absences), workload redistribution, or changing care needs. By contrast, hospital level factors often elicited skepticism or frustration. Typically involving policy or financially driven decisions, like not reserving a bed for short-term ICU transfers or implementing “bed-for-bed” policies, these were perceived as less justified and externally imposed. Yet, despite this disapproval, these impositions were rarely challenged directly.

Personal considerations also played a decisive role in whether contact was maintained or disrupted. Nurses described a common pattern: after two to three shifts with the same patient, they often preferred to rotate. Two primary reasons were cited for this: nurses claimed delivering care for the same patient as “boring,” or they attributed their preference to physical or mental workload. But when probed further, nurses often pointed to relational dynamics as underlying motivator. Patients perceived as unfriendly or difficult to connect with triggered a desire for reassignment. If this difficulty was felt individually, it could evoke feelings of inadequacy or failure; when shared by the team, it led to solidarity. Conversely, nurses also intentionally broke continuity when they felt emotionally involved, fearing professional vulnerability as the relationship deepened. Notably, some participants mentioned that emotionally intense cases were remembered and discussed years later, underlining their profound impact.

### 3.3 Synthesis of findings

The integration of findings shows a clear congruence between authorship patterns and the experiences reported by nurse. While the quantitative results demonstrate a sharp decline in recurrent contact after the second shift, the qualitative insights reveal how organizational realities and personal considerations together shape this pattern.

These combined findings highlight a notable discrepancy: despite recognizing sustained contact as both a professional value and a clinical necessity, a shared climate of acceptance allows fragmentation to persist without significant visible tension. At the organizational level, disruptions are often viewed as unavoidable, resulting in an environment where relational continuity is seldom prioritized. At the individual level, nurses identify a specific two-to-three-shift threshold where the preference for rotation increases due to the emotional demands of sustaining contact. This alignment suggests that organizational constraints and mechanisms of relational regulation effectively converge; disruptions are rarely contested because they coincide with the nurse's need for relational distance after two to three shifts. Together, these factors explain the limited realization of relational continuity in daily practice.

## 4 Discussion

This study demonstrates that the structural preconditions for relational continuity, operationalized as recurrent nurse–patient contact, are only limitedly present in clinical practice. Although nurses describe caregiver continuity as a professional value, our findings reveal a persistent gap between this value and daily practice. This aligns with hospital studies spanning several decades (Bostrom et al., 1994; Tonkikh et al., 2020; Yakusheva et al., 2017), suggesting that limited relational continuity remains a pervasive feature of hospital care. By synthesizing qualitative and quantitative findings, our study offers an explanatory framework that clarifies the underlying mechanisms and contextual factors that shape the realities of relational continuity.

Nurses in our study frequently invoke organizational arguments, such as scheduling or workload distribution, to explain disruptions in continuity. This focus is consistent with nursing literature, where continuity is described as a key organizing principle in care delivery models designed to facilitate sustained nurse–patient contact (Geltmeyer et al., 2024). However, while efforts to improve work organization typically focus on staffing models or scheduling adjustments, caregiver continuity itself is rarely examined as a variable, even in large-scale studies such as the influential RN4CAST projects (Aiken et al., 2012; Ausserhofer et al., 2014). Yet, our findings show that disruptions in continuity cannot be attributed to organizational constraints alone. Even when nurses possess the organizational agency to assign their own patients, they make deliberate choices that limit sustained contact, utilizing their autonomy to prioritize other considerations over continuity. Our findings point to a tacit, unanimous acceptance of these disruptions; the fact that a patient encounters yet another different nurse is rarely questioned. This unit-level consensus effectively shields such practices from professional scrutiny. Consequently, despite the organizational agency to act otherwise, nurses operate within a normalization of practice where the professional value of relational continuity remains unfulfilled. This challenges the linear assumption that organizational interventions alone will naturally result in higher continuity.

While organizational factors do impose real limitations, they do not fully explain nurses’ decisions regarding continuity. Ultimately, these choices appear to be also shaped by the emotional demands of sustained patient contact. A notable finding of our study is the emergence of a 'two-to-three-shift threshold': nurses are generally willing to maintain continuity during two to three shifts with the same patient. This is particularly true when social rapport is established, making this initial period both rewarding and manageable. Beyond this window, however, nurses tend to withdraw from ongoing contact, even when they retain control over patient assignments. This pattern is intriguing, as literature indicates that nurse–patient relationships often deepen after the initial days, enhancing understanding of patient needs, fostering trust, and improving care outcomes (Allande-Cussó et al., 2022; Dinç and Gastmans, 2013; Lotzkar and Bottorff, 2001). Yet, when faced with the emotional intensity of sustained contact beyond this threshold, nurses in our study utilized their organizational agency to disrupt continuity. Although they consistently emphasized the benefits of continuity in principle, they relied on pragmatic rationalizations, citing organizational constraints or team considerations, to justify these disruptions. Notably, we found no explicit evidence of moral distress; nurses accommodated disruptions without apparent inner conflict. This suggests that they circumvent the moral conflict associated with falling short of the relational ideal by framing disruptions as inevitabilities. In this light, rationalizations serve as a protective mechanism where organizational constraints and the drive for self-protection functionally converge. This creates a mutually perpetuating dynamic: because these disruptions offer a functional benefit, nurses are unlikely to challenge the organizational practices that facilitate them. Consequently, the narrative of inevitability effectively shields the emotional demands underlying the decisions to disrupt continuity from critical reflection.

Ultimately, these findings suggest that improving relational continuity requires more than just organizational adjustments; they point to a need for fundamental recognition of the emotional manageability of sustained contact. Strategies could integrate organizational interventions with a focus on the relational realities of practice, acknowledging that addressing these underlying drivers may be essential to bridging the gap between professional values and clinical reality.

## 5 Strengths and limitations

A strength of this study is its explanatory sequential design, which enabled us to understand the reasons behind the quantitative results on low continuity. Another strength is the explicit focus on relational continuity from the perspective of nurses, an angle that is underrepresented in existing literature. By foregrounding nurses’ experiences and the relational dimensions of care, this study contributes to a more nuanced understanding of continuity of care in acute hospital settings.

Some limitations must also be acknowledged. One limitation is that the quantitative proxy for relational continuity, recurrent contact based on authored reports, captures the structural prerequisite of this concept rather than its relational quality. Consequently, this proxy reflects the opportunity for relationship building, but does not guarantee the actual development of a therapeutic connection that defines relational continuity. Additionally, the qualitative phase may have been subject to self-selection bias if recruited nurses had a stronger relational orientation. However, the prominence of withdrawal patterns even within this group suggests a deep embedment in clinical practice. Finally, our selection criteria for patient stays (9 to 42 shifts) likely introduced selection bias, as hospital admissions are typically right-skewed with a high frequency of short stays and a 'long tail' of extended hospitalizations. By excluding both ends of this spectrum, our findings should be interpreted as representative of average-length clinical admissions.

## 6 Implications for practice and policy

Based on our findings, efforts to foster relational continuity could benefit from a dual perspective. While organizational adjustments, such as continuity-focused patient assignments, create the necessary structural conditions, our data suggest that the relational and emotional realities also play a significant role in nurses’ decision-making. Addressing these interpersonal factors alongside structural changes might be important for supporting continuity in similar acute care settings.

Furthermore, the patterns observed in this study invite reflection on how nurse–patient relationships are shaped in contemporary hospital care. Given the 'two-to-three-shift' threshold, striving for extended nurse–patient contact may not always be feasible or desirable for every nurse.

In this context, exploring the potential of short-term but meaningful connections could offer a more realistic basis for achieving relational engagement that is both impactful for the patient and emotionally sustainable for the nurse.

## 7 Future research

This study explored why relational continuity often remains limited in hospital nursing, focusing on the underlying mechanisms rather than clinical effectiveness. Future research could further examine the interplay between organizational constraints and the emotional dimensions of care, incorporating patients’ perspectives to understand how the observed two-to-three-day pattern affects relationship building. Intervention studies are needed to identify strategies that address the emotional labor and pragmatic rationalizations identified here as barriers to relational continuity.

Another key area for inquiry is whether individual continuity is structurally and emotionally feasible in high-acuity environments, or whether team-based continuity models offer a more realistic way to reconcile professional values with clinical realities. Once these relational foundations are better understood, longitudinal, multi-site designs could more effectively examine the impact of relational continuity on patient, nurse, and organization-level outcomes.

## 8 Conclusion

As hospital stays shorten and care needs intensify, continuity in nursing care is both increasingly challenging and increasingly important. This study suggests that relational continuity is currently limited, driven by an interplay between organizational constraints and nurses' own tendency to limit recurrent contact. By identifying these dynamics, this study sheds light on why relational continuity remains fragile. It indicates that sustainable improvement requires a dual focus: addressing organizational constraints alongside the emotional demands and relational realities nurses experience during sustained patient contact.

## Declaration of generative AI and AI-assisted technologies in the manuscript preparation process

During the writing of the original draft, the first author used ChatGPT (OpenAI, GPT-5) in order to provide phrasing suggestions and enhance text clarity. The first author reviewed and edited all content as needed and takes full responsibility for the published article.

## Data availability statement

The data that support the findings of this study are available from the corresponding author upon reasonable request.

## Funding sources

This research received no specific grant from any funding agency in the public, commercial, or not-for-profit sectors.

## Ethics approval statement

This study did not fall under the Medical Research Involving Human Subjects Act, as confirmed by METC-LDD [non-WMO approvals Z19.053 and N23.019]. The research complied with the Code of Ethics for the Social and Behavioral Sciences (2018) and the Dutch General Data Protection Regulation (AVG). All participants provided written informed consent prior to focus group participation.

## CRediT authorship contribution statement

**Heleen van Erp:** Writing – original draft, Visualization, Validation, Project administration, Methodology, Investigation, Formal analysis, Conceptualization. **Jet Bussemaker:** Writing – review & editing, Supervision, Conceptualization. **Meralda Slager:** Writing – review & editing, Supervision, Formal analysis, Conceptualization. **Janneke de Man-van Ginkel:** Writing – review & editing, Validation, Supervision, Methodology, Formal analysis, Conceptualization.

## Declaration of competing interest

The authors declare that they have no known competing financial interests or personal relationships that could have appeared to influence the work reported in this paper.

## Appendix Supplementary materials

Image, application 1
