# Supplementary material for: Relational continuity in nurse–patient contact on hospital wards: An explanatory sequential mixed-methods study
Source: Int J Nurs Stud Adv. 2026 May 25;11:100560. doi: 10.1016/j.ijnsa.2026.100560 (PMC13226210; doi:10.1016/j.ijnsa.2026.100560)
Supplement: Supplementary file 1 [file mmc1.docx]

**Appendix A: Focus group topic guide; observational protocol; debriefing protocol**

*Note: This guide is a translation of the original [language] version used during data collection.*

**TOPIC GUIDE focus groups**

**Focus group protocol**

**Participants [coded names nurses and wards]:**

**1**

**2**

**3**

**4**

**5**

**6**

**Date and time focus group:**

**Moderator:**

**Note-taker:**

| **Topic** | **Question** |
| --- | --- |
| Figures from quan study | ***Nursing Documentation and Continuity***  *Introduction based on visual map*  Initial question: Hearing this, what are your thoughts? Could you react to these findings?  Scenario A: If participants are not surprised  *Probe:* I noticed that this doesn't surprise you; why is that? Could you explain that to me?  *Probe:* How does this frequency affect the continuity of your contact with patients?  *Probe (if they find it unimportant):* What makes this unimportant in your view? What do you focus on instead?  Scenario B: If participants are surprised  *Probe:* How does this work in daily practice?  *Probe:* How are patient assignments determined on your ward? How much influence do you have on this process? What are your thoughts on that? |
| Patient assignment process | Initial questions (current practice):  Could you describe how it is determined which patients you are assigned to on your ward?  For example, how did that process go this morning when you arrived for your day shift?  Influencing factors and continuity:  What factors influence whether or not you will be assigned to the same patient again tomorrow?  Does your own preference play a role in this (i.e., whether you *want* to be assigned to them)?  What are the barriers to achieving your preferred assignment? Conversely, what factors facilitate it?  How do you feel about this process? Would you like to see it handled differently?  Satisfaction and preferences:  Could you describe a situation where you were satisfied with the assignment? What made it work?  On the other hand, when are you dissatisfied with the assignment? Why is that?  Which criteria do you consider important when creating the assignment? Of those, which is the most critical?  Agency and history:  Have you ever protested against an assignment? If so, what were your reasons?  How much weight is given to "continuity of care" (i.e., whether you have cared for that specific patient before) when making the assignment for a shift? |
| Impact of continuity on patient and nurse | Impact on the patient:  In your opinion, what is the effect of "continuity of care" (having the same nurse) on the patient?  Can you provide examples of how a patient benefits from this?  Are there also situations where continuity might actually be disadvantageous for a patient?  Impact on the nurse:  What is your stance on providing care to the same patient over an extended period?  Are there downsides to being assigned to the same patient too often?  *Probe (professional):* Looking at it through a professional lens, what are the implications for your role as a nurse?  *Probe (human):* And looking at it as one human being caring for another, how does it affect you personally?  The professional relationship:  How does continuity influence the relationship between you and the patient?  Does it change the quality of the information you receive or the nursing care you provide? |
| Differences between initial and subsequent shifts | The first encounter:  Could you tell me about the interaction with a patient when you are assigned to them for the first time?  Repeated contact:  What is it like when you are assigned to the same patient for a third or fourth time?  What exactly is different in those instances? What does that mean for you personally and professionally?  Is there a difference between caring for a patient you’ve just met versus one you have cared for multiple times?  What exactly is that difference? After how much time (or how many shifts) do you begin to feel "familiar" or "comfortable" with a patient?  Specific reflection:  For example, if you are standing at the bedside of the same patient for the third consecutive day: what is that experience like for you? |
| Turning point | Perceptions of repeated assignments:  What is your overall perspective on being assigned to the same patient multiple times?  At what point do you experience being assigned to someone as "(too) much"? Where do you draw the line?  Benefits and challenges:  What do you find rewarding about caring for the same patient over a longer period?  On the other hand, do you ever find it difficult or even frustrating to be assigned to the same patient again? What exactly makes it frustrating at that moment?  In which situations do you consider it an advantage, and when is it not?  Concrete examples (The "small moments"):  Could you share an example of a "small positive moment"—a time when you thought: *"I’m glad I’m assigned to this patient again"*?  Conversely, could you describe a "small irritation"—a moment when you thought: *"Oh no, not again"*?  Influencing factors:  What role does the patient play in these feelings? (e.g., the severity of their illness, their personality, or the involvement of their family/next of kin). |
| Team dynamics and ward culture | Peer requests:  Do colleagues ever specifically request to be assigned (or *not* to be assigned) to the same patient again?  Social norms and ward culture:  How is such a request generally received by the rest of the team?  What is the prevailing attitude toward these requests?  To what extent is it socially accepted within the team to express a personal preference for patient assignments? |
| Differences between shifts (day, evening, night) | Shift Dynamics:  Is there a difference in the interaction with patients when you are working a day shift versus an evening shift?  What exactly causes the interaction to change between these shifts?  Comparative Reflection:  I notice that for [Participant X], the shift type significantly changes the contact, whereas for [Participant Y], it doesn't seem to make a difference. Could you both elaborate on that?  What factors explain these different experiences? (e.g., the pace of the ward, medical rounds, or the presence of family). |
| *Closing* |  |
| Closing question_Content | Have you had the opportunity to say everything you wanted to say today?  Are there any other points you consider important for me to take away from this discussion? |
| Closing question_Process | Do you have any questions regarding the interview or the study in general?  How did you experience the interview today? Looking back on our discussion, what are your thoughts on how it went?  Is there anything you would like to add regarding the process or the topics we discussed? |
| Final closing & gratitude | ***Concluding the Session***  Summary and purpose:  To bring us back to the purpose of today's meeting: [brief summary of main subjects]. Does this accurately reflect what we have covered today?  Validation (member check):  I want to ensure I’ve understood you correctly. Please feel free to correct me if I’ve missed anything or misinterpreted a point, as I want to make sure I don't leave with the wrong impressions.  Expression of gratitude:  Thanking participants, giving small gift |
|  |  |
| In subsequent focus groups | Validating previous findings:  In previous focus groups, participants mentioned that […]. What are your thoughts on this?  Earlier groups suggested that […]. To what extent do you recognize this in your own experience?  Clarifying ambiguity:  We have heard several statements regarding […], but we don’t yet fully understand the exact meaning behind them. What is your interpretation of this?  The term [...] was mentioned in earlier sessions, but its practical implication remained unclear. Could you help us clarify what is meant by this in a clinical setting? |
| General probes | Staying close to own curiosity, for example: I hear you mentioning [X], and it intrigues me. Could you dive a bit deeper into that? What exactly makes you highlight this specific point, rather than something else? I'm curious: what is the core reason that this particular aspect stands out to you?  Bringing it back to the group: Hearing [Participant X] say this, how would the rest of you respond? What does that trigger for you? What does that mean for you personally when you hear a senior colleague describe it this way? Could you build on what was just said? I’d love to hear your perspective on that? Does this resonate with you? Could you tell us more about your own experience with this?  Silence (approx. 5 seconds)  General encouraging signals  Immediate probing questions:  And then?  That’s interesting; could you tell me a bit more about that?  I’m curious, how does that work in practice?  How do you feel about that? / What are your thoughts on that?  What does that mean to you?  Is that the most important aspect for you?  What do you mean by [specific term]? Could you give an example?  Could you help me better understand what you just said?  I’m not sure if I fully follow you; could you help me understand what it was like to experience that / to be in that situation?  What was it that made you feel that way?  Immediate clarification:  Requesting details  Exploring the 5 W’s and how  Probing on three levels:  What did you do?  What were you thinking?  What did you feel? |
| Back-up strategies | *Note: These strategies were included in the protocol to stimulate discussion if needed, though they were not required during the sessions.*  **Inversion**: ==Provide a thorough explanation of the method==  Prompt: "Imagine you wanted to report on the same patient as *infrequently* as possible. How would you go about that?"  **Cartooning**:  Prompt: "What are these two people saying to each other?"  **Ideal scenario:**  Prompt: “Imagine you are the hospital director tomorrow. Which two measures would you implement regarding the contact moments between nurses and patients?” |

| **Observational data protocol**  Spatial mapping: draw diagram of the setting, including table arrangements and numbered seating.  Temporal logging: assign a time stamp to each observation. |
| --- |
| **Observational Focus Points**  Social dynamics  Manner of expression: How do participants convey their messages? What is the tone and delivery?  Linguistic patterns: Which specific language is used? (e.g., metaphors, imagery, or mitigating language/euphemisms).  Emotional intensity: What is the perceived intensity of the emotions associated with this specific topic?  Group interaction and norms: Response to relational continuity: What occurs within the group dynamic when the subject of relational continuity is introduced?  Sanctioning of opinions: Are participants socially "punished" or silenced for expressing a particular viewpoint?  Group consensus and influence: Does the group tend to follow the lead or opinion of a specific nurse?  Social desirability: Which opinions or perspectives appear to be more socially accepted or dominant within the group? |
|  |

**Debriefing protocol moderator and note-taker – directly after each focus group**

What are our first impressions of the session? How would we describe the overall atmosphere?

Did the discussion flow naturally, or did it feel strained or fragmented?

To what extent do we feel the participants provided honest and candid responses? Were they open, or did they seem to be 'evading' certain topics?

Did everyone have an equal opportunity to contribute, or were some voices dominant?

Were there any specific disruptive factors during the session (e.g., interruptions, environmental noise, time pressure)?

What were the core ideas and themes that emerged? Which statements were most striking or unexpected?

Were there any findings that surprised us, or new questions that arose?

How did this session differ from previous groups?

Which topics require further exploration in the next sessions?

Are there any necessary adjustments to the moderation style or the topic guide for the next group?

Are there any other noteworthy observations or remarkable incidents?
